# A comprehensive urban programme to reduce energy poverty and its effects on health and wellbeing of citizens in six European countries: study protocol of a controlled trial

**DOI:** 10.1186/s12889-022-13968-2

**Published:** 2022-08-19

**Authors:** Merel Stevens, Hein Raat, Maite Ferrando, Beatriz Vallina, Rebeca Lucas, Lucie Middlemiss, Josep Rédon, Elena Rocher, Amy van Grieken

**Affiliations:** 1 Department of Public Health, Erasmus University Medical Center, P.O. Box 2040, 3000 CA Rotterdam, the Netherlands; 2 Kveloce I+D+i, 46002 Valencia, Spain; 3 Sustainability Research Institute, School of Earth and Environment, University of Leeds, Leeds, UK; 4 INCLIVA Research Institute, University of Valencia, Valencia, Spain; 5 Las Naves, 46024 Valencia, Spain

**Keywords:** Energy poverty, Social-ecological model, Health-related quality of life, Health inequity

## Abstract

**Background:**

Nearly 11% of the European population is affected by energy poverty. Energy poverty is defined by the European Commission (2016) as the inability to afford basic energy services to guarantee a decent standard of living. Energy poverty is considered a complex, multidimensional problem that affects environment, housing, urban development, and health. Living in energy poverty conditions is associated with poorer human health and wellbeing. Hence, the WELLBASED intervention programme aims to design, implement and evaluate a comprehensive urban programme, based on the social-ecological model, to reduce energy poverty and its effects on the citizens’ health and wellbeing in six European urban study sites: Valencia, Spain; Heerlen, The Netherlands; Leeds, United Kingdom; Edirne, Turkey; Obuda, Hungary, and; Jelgava, Latvia.

**Methods:**

A controlled trial is performed. A total of 875 participants are recruited (125–177 per study site) to receive the WELLBASED intervention programme for 12 months (intervention condition) and 875 participants act as controls (control condition). Data will be collected with a baseline measurement at inclusion (T0), and follow-up measurements after 6 months (T1), 12 months (T2), and 18 months (T3). In both study arms, effects of the WELLBASED intervention programme are measured: health-related quality of life (HR-QoL), mental health, frailty in older adults, self-perceived health, chronic conditions, and care utilization. At the same time points, household expenditure on energy and energy consumption are obtained. In the intervention arm, health-monitoring data (i.e. peak flow, oxygen saturation, blood pressure, and heart rate) are obtained monthly and sleep quality with a three-month interval. Household data with regard to temperature, humidity and air quality are collected near real-time by home sensors. Qualitative interviews are conducted in each study site to evaluate the impacts of the WELLBASED intervention programme and to help explain findings.

**Discussion:**

The WELLBASED intervention programme will provide new insights into the effectiveness of a comprehensive urban programme to tackle energy poverty and its effects on health and wellbeing across Europe. Hence, this study can contribute to European-wide replicable solutions for policy-makers and city practitioners to alleviate energy poverty.

**Trial registration:**

ISRCTN registry number is ISRCTN14905838. Date of registration is 15/02/2022.

**Supplementary Information:**

The online version contains supplementary material available at 10.1186/s12889-022-13968-2.

## Background

Energy poverty has become a major societal challenge that is estimated to affect nearly 11% of the European population [1, 2]. Due to rising energy prices, low incomes and poor energy efficiency of housing, around 54 million Europeans are not able to adequately heat their homes at an affordable cost [3, 4]. Energy poverty is defined by the European Commission (2016) as ‘’the inability to afford basic energy services (heating, cooling, lighting, mobility and power) to guarantee a decent standard of living due to a combination of low income, high energy expenditure and low energy efficiency of homes’’ [5]. It is most likely to occur in urban areas with high unemployment and poverty along with poor quality buildings or poor built environment [3].

A growing evidence base highlights the negative impact of living in energy poor conditions on human health and wellbeing [6–11]. For example, a survey among 536 United Kingdom social housing residents reported that the inability to keep a households’ living room warm together with concerns about the affordability of energy bills, was associated with lower satisfaction with life [9]. Residing in cold homes has been associated with poorer mental health, cardiovascular and respiratory diseases and minor illnesses such as cold and flu [9–12]. Those with chronic and severe illnesses, including disabilities, and older people seem to be worst affected [13–15]. This is explained by the fact that sedentary or ill people are less able to generate their own heat, spend more time inside their homes, and are more likely to be on low incomes and thus unable to afford adequate energy [11, 16, 17].

Interventions to reduce energy poverty and mitigate its effects on health and wellbeing are needed. Thus far, the majority of studies reporting on interventions to reduce energy poverty focus on one specific intervention strategy; [6, 18–21]. that is the implementation of housing improvement measures [6, 19–21]. Although, the improvement of housing, e.g. thermal comfort in the home, can lead to health improvements [19], a more comprehensive intervention approach seems warranted [22, 23]. Such an approach may be guided by the social-ecological model, which maps the interactions between the individual, the community, and the physical, social, and political environments that affect health and wellbeing [24]. The purpose of this paper is to describe the evaluation framework for the WELLBASED intervention programme: a comprehensive, urban programme to improve health and wellbeing by tackling energy poverty.

### WELLBASED

The WELLBASED project will design, implement and evaluate a comprehensive urban programme, based on the social-ecological model, to reduce energy poverty and its effects on the citizens health and wellbeing. Six urban study sites in Europe (Valencia, Spain; Heerlen, The Netherlands; Leeds, United Kingdom; Edirne, Turkey; Obuda, Hungary, and Jelgava, Latvia) will implement the WELLBASED intervention programme. The WELLBASED study cities represent not only different urban realities but also a diverse range of welfare and healthcare models. The WELLBASED project was established in response to the HORIZON 2020 call “Innovative actions for improving urban health and wellbeing- addressing environment, climate and socioeconomic factors’’, funded under GA 945,097.

### Objectives

The aim of this study is to evaluate the WELLBASED intervention programme, using a controlled trial design. The short, and mid-term effects of the programme on energy poverty and health and wellbeing indicators are evaluated in comparison to a control condition (i.e. no specific programme to address energy poverty is applied). Cost-effectiveness of the intervention programme is assessed. Furthermore, differential impact of the intervention programme with regard to gender and social determinants is explored. Additionally, interviews and focus groups are implemented in each study site to evaluate the impact of the intervention programme and to help explain outcomes. To this end, the controlled trial design is combined with a qualitative approach that offers explanations of the impact and implementation of the WELLBASED intervention programme.

### Hypothesis

The hypothesis of this study is that vulnerable people living in energy poverty who participate in the WELLBASED intervention programme have more favourable results with regard to indicators of energy poverty, health and wellbeing in comparison to the people participating in the control condition.

## Methods

This study protocol adhered to the SPIRIT guidelines for clinical trial protocols [25].

### Study design

A controlled trial is performed with an intervention condition and a control condition (i.e. no specific programme to address energy poverty is applied) in six study sites in Europe. Measures are taken at baseline (T0), at 6 months (T1), at 12 months (T2) and 18 months after baseline (follow-up; T3) in both research groups. In addition, in the intervention condition additional frequent monitoring of health and housing conditions takes place. Moreover, qualitative interviews with a subsample of participants undergoing the intervention condition take place near the beginning of the intervention (3–6 months) and after termination of the intervention (15–18 months).

The WELLBASED intervention programme targets adults ≥ 18y in vulnerable situations, living in energy poverty conditions that belong to one of the study sites. An overview of study sites and the target population is presented in Table 1. Local organisations (e.g. the social services department or an non-governmental organisation (NGO) that supports vulnerable groups) or the local research team, depending on study site, select eligible individuals. Recruitment of study participants and baseline data collection is scheduled to commence in autumn 2022. Potential participants in the catchment area of an intervention or control site receive information about the study and an invitation to provide informed consent to join the study by the study site team. Generally, in each selected household, the person who is considered responsible for the accommodation or best placed to provide the information is invited to join the study and to complete questionnaires. This participant is also invited to participate in health data collection and, when applicable, to join qualitative interviews at a later stage. In some cases more than one person per household may be invited to join the study. Participants only join the study after they have provided informed consent. Study participants, intervention providers and research assistants are not blinded due to the nature of the intervention. The study is performed in accordance with the capacity, organizational and contextual factors of each of the six participating cities, as described below.

**Table 1:** Study site characteristics

STUDY SITE	RECRUITMENT OF PARTICIPANTS	
	**TARGET GROUP**	**NUMBER OF PARTICIPANTS**
VALENCIA (SPAIN)	Three districts with high sociodemographic vulnerability, due to an aged population, lower incomes than the city average and higher percentages of people at risk of poverty	177 participants per study arm
HEERLEN (THE NETHERLANDS)	Social housing tenants from two districts in the northern part of the city with low incomes, high energy bills, low energy measures and bad housing conditions	156 participants per study arm
EDIRNE (TURKEY)	Low-income households in five neighbourhoods, where vulnerable groups, including Roma, are highly represented	125 participants per study arm
JELGAVA (LATVIA)	The most vulnerable households, described by low income levels, long-term unemployment (> 1 year), disabled people, poor housing quality, single-parent families, pensioners (especially suffering loneliness), and provided by the municipality	146 participants per study arm
LEEDS (United Kingdom)	Social housing tenants, managed by the City Council, with poor housing quality, classified as energy efficiency band D or below. Target group has different vulnerabilities: low income, older people, disabled people, single parents and recent migrants	125 participants per study arm
OBUDA (HUNGARY)	Vulnerable inhabitants of Óbuda-Békásmegyer, the 3^rd^ district of Budapest, characterised by low incomes, victims of domestic violence and/or drug abuse, households with disabled and/or chronically ill members, unemployed members, and single mothers	146 participants per study arm

### Intervention condition

Participants in the intervention condition receive the WELLBASED intervention programme. The definition of the WELLBASED urban programme follows the theoretical basis of the social-ecological model [24]. This is characterized by fixed factors (core non-modifiable factors), such as age, sex and genetics, and by a set of potentially modifiable factors expressed as a series of layers of influence. The latter includes personal lifestyle, the physical and social environment and wider socio-economic, cultural and environment conditions. The social-ecological model maps the interactions between the individual, the community, and the physical, social, and political environments that affect health and wellbeing.

A general framework of the urban programme is established and adapted to each study site. Focus groups are implemented in each study site with stakeholders and end-users to co-create the interventions, to ensure that they are carried out according to the real needs of end-users.

Actions are defined for each layer of the social-ecological model, to maximize the scope and benefits of the intervention (please see Additional file 1 for examples of potential actions for each layer in more detail):

1. Individual lifestyle factors: practices oriented to improve individual lifestyles regarding health, energy efficiency, energy costs, and residential comfort (e.g., individual energy advice).
2. Social and community networks: activities oriented to strengthen communities, mainly those oriented to promote community support and mutual aid, and therefore moving from an individual to a collective support approach (e.g., energy cafes).
3. Living and working conditions: practices oriented to improve the access to dignified living conditions, supporting comfortable and healthy homes (e.g., fuel debts support, improved energy efficiency).
4. General socio-economic, cultural and environmental conditions: practices and policies that aim to make structural changes on the socio-economic context, mainly referring to both energy and to household policies (e.g. observatories).

### Control condition

In the control condition the usual activities of participants continue. No intervention actions are undertaken. Some study sites use a waitlist design to allow participants in the control condition to receive the intervention programme after data collection has terminated. Study sites might provide incentives to control participants such as money, groceries, keeping of study devices after completion of the data collection.

### Study population and eligibility to participate in the study

All participants who provide informed consent are enrolled in the study. We aim to include 1750 participants in total. Each study site allocates at least 125 participants to the intervention condition and 125 participants to the control condition. Detailed numbers of participants per study site are presented in Table 1. Study sites might apply randomization at individual or cluster level to create intervention and control condition.

Study participants are included if they: (i) are aged ≥ 18 years old, (ii) are in a vulnerable situation (e.g. unemployed, low income, single parents, parents with dependent children, seniors (65 +) with dependency conditions, seniors (65 +) living alone, people with disabilities attended by social services, belonging to a minority, migrant situation), (iii) living in energy poverty conditions, and (vi) belonging to the recruitment sites identified by the local partners for the study. The research assistant recruits potential participants in collaboration with local agencies that work in the catchment area. Together they determine energy poverty conditions (criterion iii), based on their knowledge of and experience with the target population. The primary indicators suggested by EU Energy Poverty Observatory (EPOV) are used as guideline to assess the energy poverty status. EPOV established the following criteria:

i. arrears on utility bills;
ii. low absolute energy expenditure (below half of the national median (M/2));
iii. high share of energy expenditure in income (double of the national median share of energy expenditure in income (2 M)) and
iv. inability to keep home adequately warm [26].

Persons are not eligible to participate if they have previously been beneficiaries of a previous similar intervention, cannot adequately participate in the intervention actions proposed in the study site (e.g. intellectual disabilities, severe language limitations) or are illegally connected to the electricity grid.

### Data collection

Data are collected using complementary methodology: self-reported questionnaires on health, wellbeing, household energy expenditure and consumption, health monitoring (e.g. blood pressure measurements), household data from sensors, and qualitative data on the lived experience of the intervention. A data platform, established prior to the start of the study, will support data collection and integration of data from different sources.

#### Data collection in both study arms

Individual level data on health, wellbeing and energy-poverty indicators in both research groups are collected through questionnaires completed by the participating adults (self-report). The questionnaires can be completed on paper or digitally through a secured mobile or web-based application. Instruments for which no validated translations are available will be translated (forward and backward translations). Before the start of the study, the questionnaire is pilot-tested to ensure its user-friendliness in terms of appropriateness, comprehensibility and length.

Instruments and variables collected in both study arms are presented in Table 2.

**Table 2:** Instruments and variables collected in both study arms

Variable	Instrument/indicator	Data source	Timeline
**Sociodemographic details: age, sex, gender, occupation, etc**	Ad-hoc questionnaire	Online/offline self-reported questionnaire	Baseline
**HEALTH AND WELLBEING MEASURES**			
**Quality of life**	EQ-5D-5L [27]	Online/offline self-reported questionnaire	Baseline, 6, 12 and 18 months
**Mental health: depression, anxiety, stress**	Depression Anxiety Stress Scales 21 (DASS-21) [28]		
**Frailty**	Self-Administered Multidimensional Prognostic Index Short Form (SELFY-MPI-SF) [29]		
**Subjective comfort in households**	European Statistics on Income and Living conditions survey (EU-SILC) [30]		
**Comorbidities**	International Consortium for Health Outcomes (ICHOM) Overall Adult Health set [31]		
**Lifestyle behaviour: Body Mass Index (BMI), alcohol consumption, smoking status**	ICHOM Overall Adult Health set [31]		
**Lifestyle behaviour: Physical activity**	One item of the Internal Physical Activity Questionnaire (IPAQ) [32]		
**Loneliness**	University of California, Los Angeles (UCLA) 3-item Loneliness Scale [33]		
**Control over life and social support**	Adult Social Care Outcomes Toolkit [34]		
**Health care use**	Self-management resource center (SMRC) Health Care Utilization questionnaire [35]		
**Decompensation of chronic disease**	Number of diagnosed exacerbations, all health settings (emergencies, acute units, hospitalization, primary care)	Online/offline self-reported questionnaire (if available, direct extraction from Electronic Health Records)	Baseline, 6, 12 and 18 months
**Readmissions**	Admissions to the emergency department, acute units or regular hospitalisation		
**Use of primary attention services**	Visits to the primary attention services distinct from those aimed at renewing the prescriptions		
**ENERGY POVERTY RELATED MEASURES**			
**Energy poverty indicators**	European Statistics on Income and Living conditions survey (EU-SILC) [30]	Online/offline self-reported questionnaire	Baseline, 6, 12 and 18 months
**Attitudes/coping**	Self-reported scale, three items of the European Social Survey [36], two items of the Climate Change Worry Scale [37]	Online/offline self-reported questionnaire	
**ENERGY EFFICIENCY EVALUATION**			
**Energy consumption**	On grid (e.g. electricity, gas, heat) and off-grid (e.g. bottled gas, coal) energy consumption	Online/offline self-reported questionnaire, if possible, smart meters or energy provider	Baseline, 6, 12 and 18 months
**Household income spent on different sources of energy**	% of income/Euros	Online/offline self-reported questionnaire and, if possible, energy provider	

The main outcome is the change in health-related quality of life (HrQoL). This is measured by the EuroQol 5-level EQ-5D (EQ-5D-5L) instrument [27]. Secondary outcome measures include satisfaction with life, mental health, outcomes related to lifestyle behaviour, comorbidities, loneliness, subjective comfort in households, control over life, and social support. Frailty is assessed in older adults. Energy-related outcome measures are energy poverty indicators as defined by EPOV [26], attitudes towards adoption of energy efficiency measures, and coping behaviours.

Additional data on health care use are collected through electronic health records (when possible) for both intervention and control condition. This entails visits to the emergency ward and relevant care use outcomes. If data is not available through the electronic health records this information is obtained in the self-reported questionnaire.

Household spending on energy and energy consumption is obtained for participants in the intervention and control condition via the self-reported questionnaire. Depending on the study site additional data may be collected via smart energy meters or via energy providers.

#### Data collection in intervention condition

In the intervention condition, additional data will be collected through health monitoring. Peak flow, oxygen saturation (SpO_2_), blood pressure and heart rate are measured every month through the use of health monitoring devices. Devices, such as Fitbit-like trackers, send health data to the data platform directly. In other instances, data is obtained manually during a home visit. Peak flow and SpO_2_ are measured in a resting position and after a six minutes’ walk. Blood pressure and heart rate are measured three times in a resting position with a three-minutes interval. Sleep quality is measured with the Pittsburgh Sleep Quality Index with a three-month interval [38].

Home sensors collect household data on temperature, humidity, and air quality in the intervention condition. Data are collected near real-time.

Qualitative interviews are conducted in each study site with 20 subjects of the intervention to evaluate the impacts of the intervention and to help explain findings. The qualitative research is longitudinal: the first interview takes place after the project has started, and the second when the project is finished. Participants are asked in the baseline questionnaire if they are willing to be involved in the qualitative part of the study. From these volunteers, the baseline questionnaire results are used to construct a sample which reflects the diversity of the main groups identified. Specific attention is paid to ensuring a gender-balanced sample, and to including people who report a range of health and energy poverty experiences. General guidelines for the set-up of the interviews are designed collaboratively across the research team, and training and support in implementation is offered by the lead qualitative partner. Study partners conduct interviews in their study sites and collect qualitative data from the studys’ participants following a consistent but adaptable methodology. Key topics in interviews include the participant’s experience of their home and energy use in the home, their coping strategies in the home, the wider effects of energy poverty and the impacts of the intervention.

Instruments and variables collected in the intervention condition are presented in Table 3.

**Table 3:** Instruments and variables collected in intervention arm

Variable	Instrument/indicator	Data source	Timeline
**Respiratory & cardiovascular function indicators**	Peak flow	Internet of Things (IoT) home health control devices	Monthly (30 days)
	SpO_2_		
	Blood pressure		
	Pittsburgh Sleep Quality Index [38]		Every 3 months
**Household conditions: temperature**	Celsius Degree	IoT DT home sensors	Real time monitoring
**Household conditions: humidity**	% Relative humidity		
**Household conditions: air quality**	CO_2_ and CO concentration		
**Qualitative interviews**	Impressions, comments, experience and subjective perceptions captured in focus groups and interviews & codified	Qualitative analysis codified records	3–6 months, 12–15 months

#### Additional measures

City-level data on air quality, weather, climate, green spaces, and pollution are collected through available open data.

#### Evaluation of implementation

To evaluate the implementation of the WELLBASED programme, the Reach, Effectiveness, Adoption, Implementation, and Maintenance (RE-AIM) framework is adopted [39]. Indicators such as reach (number and proportion of individuals willing to participate), adoption (number of intervention agents willing to initiate the intervention), implementation (percent of program delivery, adaptation and costs) and maintenance (long-term adaptation of the intervention) are assessed. Both qualitative and quantitative methods are applied to collect information on implementation of the WELLBASED program across study sites.

### Power consideration

WELLBASED counts with a total sample size of 1750 participants (*n* = 875 intervention group and *n* = 875 control group) distributed among the six study sites, with a minimum of 125 participants per arm per study site, as shown in Table 1. A 20% loss to follow-up (e.g. due to mortality, rehousing or impossibility to participate, as reflected in for this type of long-term studies) is expected [40]. This means the sample consists of *n* = 700 in the intervention group and *n* = 700 in the control group (total *n* = 1400) at 18-month follow-up. Equal standard deviations in the intervention and the control group are assumed, with alpha of 0.05 and power of 0.80. Thus, given six study sites with each an intervention group and control group, applying a correction factor to account for the clustered design, assuming an average cluster size of 117 citizens (1400/12) and an intra-class correlation coefficient of 0.02. For this expected overall sample size and assumptions, with regards to the continuous outcome measures (in particular, HRQoL) a difference of 0.28 standard deviation (SD) between the intervention and the control group can be detected at follow-up. This means that both at the European level, and in addition in each study site separately, small differences regarding the outcomes in the intervention group compared to the control group can be shown.

### Data management and analysis

Data from all study sites are pseudo anonymized and combined in an online data platform (handled by INCLIVA). Erasmus University Medical Center is responsible for analysis and reporting. As the project will collect health-related data, special attention is attributed to the role of each partner in terms of controllers and processors, and to the organizational and technical measures to be put in place to ensure General Data Protection Regulation (GDPR) compliance. In addition, the risks associated to data processing will be defined in the Data Protection Impact Assessment (art. 35 GDPR) to be evaluated together with the Controllers’ Data Protection Officers. The data management plan includes procedures for ensuring a high-quality data standard, according to the FAIR principles: Findability, Accessibility, Interoperability, and Reusability [41]. All data are handled confidentially and scientific data are stored anonymously.

Descriptive statistics are used to describe participant characteristics in each study site and in the total study population, and to describe implementation outcomes in the study sites. Differences between T0, T1, T2 and T3 measurements are evaluated using multilevel linear regression analyses for continuous outcome variables and multilevel logistic regression analyses for dichotomous outcome variables. Interaction tests are performed for age, gender, living situation and education level to evaluate effects in subgroups. Statistical analyses are repeated for each study site separately. We consider a *P*-value of 0.05 or lower to be statistically significant. Analyses are performed according to the ‘intention to treat’ principle. An additional per protocol analyses is performed to evaluate the impact of the dose of the intervention received on outcomes.

A preliminary cost-effectiveness analysis is performed with the baseline measurement as control condition from a healthcare perspective. The healthcare costs per individual participant are calculated by multiplying resource use (e.g., doctor appointments, hospital admissions) with corresponding unit prices if available. Utility values are calculated using the EuroQol 5-level EQ-5D (EQ-5D-5L) to estimate the quality-adjusted life years (QALYs) [27]. EQ-5D-5L has demonstrated good measurement properties such as ceiling effects, reliability, and sensitivity [42].

#### Qualitative analysis approach

During qualitative interviews households’ feedback, impressions, comments on their experience and health impacts are gathered, as well as their understandings of why things worked well or did not work out for them. These insights provide explanatory findings for the project in general, and also allows study sites to modify their activities after the baseline stage to better target those that are excluded. An iterative approach to qualitative analysis of interview data is therefore undertaken, after each round of data collection. Interview data are digitally recorded, transcribed and translated for analysis, a process led by the lead qualitative partner and drawing on the experience and expertise of key collaborators from each study site. Analysis seeks explanations of site-specific phenomena, as well as attempting to generalise about experiences of energy poor households in the face of interventions across Europe.

### Dissemination

A comprehensive dissemination and communication strategy is designed and implemented. Scientific dissemination includes papers and proceedings submitted to international conferences or peer-reviewed journals. A scientific committee supports and monitors scientific dissemination. Authorship is determined in accordance with the ICMJE authorship guidelines. Dissemination aimed at local authorities, social workers, health professionals, energy providers and the civil society (associations and NGOs) is done through the project website (https://wellbased.eu), professional magazines, congresses, fairs and (capacity building) workshops organised within the project. Networks and alliances on health and/or poverty, vulnerability or climate change are strengthened to reinforce the impact of the dissemination activities.

## Discussion

### Summary of the study aim

This study aims to evaluate the potential benefits of the WELLBASED intervention programme on several indicators of health, wellbeing, and energy poverty. The programme offers a framework of actions targeted at individual lifestyle factors, social and community networks, living and working conditions and socio-economic and environmental conditions. A pre-post controlled design is used and the WELLBASED intervention programme is implemented in six European study sites: Valencia, Spain; Heerlen, The Netherlands; Leeds, United Kingdom; Edirne, Turkey; Obuda, Hungary, and Jelgava, Latvia.

Energy poverty is highly prevalent in Europe [1, 2]. Multiple studies showed the adverse effects of living in energy poverty conditions on human health and wellbeing [7–12]. This multidimensional problem faced by many European citizens calls for a comprehensive approach to alleviate the negative impact on their health and wellbeing. Moreover, climate mitigation targets under the Paris agreement are creating further pressure to reduce household energy consumption, and fund renewable energy or energy efficiency measures. The ways in which such measures are funded can have an important impact on energy poor households: if funds are levied through energy bills this has an inequitable impact on poorer households [43]. In any case, poorer households already find it difficult to afford adequate household energy. Wealth inequality is known to affect energy consumption, and people with low incomes already spend a higher proportion of their income on energy bills [44]. This leaves them unable to afford investments in renewable energy, [6] and at risk of further vulnerability from the energy transition.

### Strengths

This study has several strengths. WELLBASED aims to implement a comprehensive intervention to fight both energy poverty and its effects on health and wellbeing across Europe. The interdisciplinary approach to the evaluation method, and comprehensiveness of data collection are a unique opportunity, creating a rich and valuable dataset on the impacts of these interventions on energy poverty and health. The development of the WELLBASED urban programme is co-created with stakeholders and end-users to ensure it serves their needs. The implementation and evaluation of the programme in different countries provides information on the generalizability and feasibility of the approach in various European settings. By utilizing a uniform questionnaire and standardized measurements, a cohesive evaluation is applied. Analysis, conclusions and recommendations drawn from mixed methods data collection during the study are rooted in the social-ecological model. This enhances insights into the impacts of particular interventions on different people, as well as the comparison of interventions and outcomes across countries.

### Limitations

We also expect to encounter some challenges. Participation of vulnerable people living in energy poverty may be challenging. The recruitment strategy seeks to involve societal partners that hold a trusting relationship with the target group. This has been shown to be an effective strategy in engaging hard-to-reach populations in health research [45, 46]. Furthermore, using the questionnaire we aim to capture the most important confounding variables to control for differences between participants in the intervention and control condition; however it remains possible that study results are subject to confounding.

## Supplementary Information

**Additional file 1:Table 4.** Potential intervention actions divided by layer of the social-ecological model.

**Additional file 2.** Draft version of participant information leaflet.

## Data Availability

The datasets used during the study are available on reasonable request by contacting the corresponding author.

## Abbreviations

BMI: Body Mass Index
EPOV: Energy Poverty Observatory
EQ-5D-5L: 5-Level EQ-5D
FAIR: Findability, Accessibility, Interoperability, Reusability
GDPR: General Data Protection Regulation
HR-QoL: Health-related quality of life
IoT: Internet of Things
NGO: Non-governmental organization
QALYs: Quality-adjusted life years
RE-AIM: Reach, Effectiveness, Adoption, Implementation, Maintenance
SD: Standard deviation
SF-12: Short-form health survey 12
SpO_2_: Oxygen saturation
