# Supplementary material for: A comprehensive urban programme to reduce energy poverty and its effects on health and wellbeing of citizens in six European countries: study protocol of a controlled trial
Source: BMC Public Health. 2022 Aug 19;22:1578. doi: 10.1186/s12889-022-13968-2 (PMC9389758; doi:10.1186/s12889-022-13968-2)
Supplement: Supplementary file 1 — Additional file 1:Table 4. Potential intervention actions divided by layer of the social-ecological model. [file 12889_2022_13968_MOESM1_ESM.pdf]

Table 4 potential intervention actions divided by layer of the social-ecological model

LAYER	EXAMPLES OF ACTIONS
• Individual lifestyle factors	• Individual energy advice<br>• Energy debt advice<br>• Optimization bill support<br>• Training to energy vulnerable people<br>• Energy audits<br>• Educational materials<br>• Energy Efficiency Toolkits<br>• Self-monitoring health Toolkits<br>• Energy monitoring through Apps
• Social and community networks	• Collective advice support<br>• Peer to peer learning<br>• Citizen assemblies<br>• Training to key actors' "identifiers" (doctors, teachers, social workers…)<br>• Volunteer on energy advice support<br>• Self-financing communities<br>• Campaigns to raise awareness

	• "Community catalysts" programmes (training and mobilising of e.g. volunteers, unemployed, students or immigrants to give energy/health services to the community)<br>• Cross-generational programmes e.g. involving youth and elderly people
• Living and working conditions	• Monetary support to energy bill<br>• Housing energy efficiency measures<br>• Renewable energy as a tool to guarantee affordable energy<br>• Fuel debts support<br>• Grants for refurbishment<br>• Employment opportunities e.g. through professional trainings on energy efficiency or rehabilitation<br>• Energy building rehabilitation<br>• Emergency alerts service (air quality, extreme temperatures)
• General socio-economic, cultural and environmental conditions	• Protection against disconnection

	• Social tariff<br>• Democratic access to energy<br>• Observatories<br>• Healthcare system protocols<br>• Access to renewable energy<br>• Manifesto and recommendations for policymakers
