# Supplementary material for: A comprehensive urban programme to reduce energy poverty and its effects on health and wellbeing of citizens in six European countries: study protocol of a controlled trial
Source: BMC Public Health. 2022 Aug 19;22:1578. doi: 10.1186/s12889-022-13968-2 (PMC9389758; doi:10.1186/s12889-022-13968-2)
Supplement: Supplementary file 2 — Additional file 2. Draft version of participant information leaflet. [file 12889_2022_13968_MOESM2_ESM.pdf]

Draft version of participant information leaflet and informed consent form (Leeds study site).

WELLBASED project, Leeds information sheets and consent forms

1. Information sheet for intervention group 2
2. Information sheet for control group 5
3. Consent form for intervention and control groups 7
4. Data Privacy Notice for intervention and control groups 9
5. Withdrawal of consent for intervention and control groups 10

DRAFT

Please note: do not make publicly available.

Draft version of participant information leaflet and informed consent form (Leeds study site).

1. Information sheet for intervention group

About this information sheet

This Participant Information Sheet is intended to help you decide if you would like to participate in the WELLBASED research study. It describes the study, what you can expect to happen if you decide to take part, and contains important information to help you make your decision. Please note that:

- Participating in this study is voluntary – it is your choice;
- If you join this study, you can change your mind and withdraw at any time;
- It is important you understand why and how this study will be conducted, please read this information sheet carefully;
- The benefits and risks of your participation are also described below.

Please take time to read this information, and if you like, discuss it with friends or family to help you decide about whether you would like to participate. Once you have read this information sheet, and if you feel you have enough information and wish to participate in the project, we invite you to give your consent to being involved by signing a consent form.

About the Wellbased project in Leeds

WELLBASED is a project aimed at improving health and reducing fuel poverty in cities in Europe, including Leeds. Fuel poverty is when people find it difficult to afford their energy bills, and it can affect people's health (including mental health), for instance, when people struggle to keep their homes warm enough in winter. The project is designed to help cities like Leeds to find ways of helping people to reduce fuel poverty and reduce the effects of fuel poverty on people's health and wellbeing.

The project involves research in six European countries (Hungary, Latvia, the Netherlands, Spain, Turkey, the United Kingdom), with households that are vulnerable to fuel poverty. In each country we will run a project to improve the lives of those vulnerable to fuel poverty, and then monitor the effects of that project on people's health and wellbeing.

In Leeds we are monitoring the effects of a Leeds City Council project to renovate buildings making them more energy efficient. Your home has been chosen to be renovated, and as such we are inviting you to be part of our study. We want to find out how the renovation affects you over the next 18 months, in particular whether it has any effects on your health, or on your ability to access energy. Please note that your building will be renovated whether you choose to be involved in our study or not.

Description of the study and your involvement

In the WELLBASED study we will monitor the effects of the building renovation on your health, wellbeing and energy consumption. The key activities we would like you to be involved in are as follows;

1. To complete a detailed questionnaire about your health, wellbeing and energy consumption on 4 separate occasions (between 01/09/2022 and 01/03/2024). We will also ask you about some key information on you and your household (e.g. age, number of occupants, income). We can help you to fill in this questionnaire if you need us to, or we can give you an internet link for you to do this yourself. This will take about 30 minutes each time and we will remind you when it needs to be done.
2. To allow us to put damp and temperature monitors in your home during the period of study (between 01/09/2022 and 01/03/2024). We will explain precisely how to do this in detail if you agree to be involved in the study, but it is a very simple process. We will send you monitors through the post, and give you clear instructions on where to place them in your home. Once you have placed the monitors around your home, they will send temperature and damp information back to us automatically.

Please note: do not make publicly available.

DRAFT

3. To allow us to collect health information on your blood pressure, heart rate, quality of sleep, peak flow and oxygen saturation. We want to visit you monthly during the next 18 months (between 01/09/2022 and 01/03/2024). Our Health Data Technician will come to your home, or to a convenient public location, making an appointment with you at a convenient time to do this.

We will also invite **some people** to participate in:

4. Two interviews about your experiences of using energy in your home, which would take place in about November 2022 and November 2023. These would take about an hour each and will feel like an informal chat about your use of energy and your health.

Risks and benefits of being in this study

Talking or answering questions about not being able to access enough energy can be upsetting, and it can also be upsetting or embarrassing to talk about your health, or your energy use with a stranger. We can make this easier for you. For instance, you can fill in a questionnaire privately online if you wish to. Information about your health and experiences will be kept private: while our research team will use the information, it will not be linked to you personally. If you are likely to be upset by thinking about and talking about these things you should probably not agree to participate in this study.

Being in this study means that we will be monitoring your health and energy use. We will try to offer additional help if you need it. This will include:

- We will offer referrals to the 'Green Doctors' scheme which offers help in reducing energy bills.
- We will also offer referrals to 'Living Leeds' which offers additional health support.
- If one of the health measurements we take is unusual we may advise you to contact your GP or go to the Accident and Emergency department. You should know that our Health Data Technician is not a qualified medical professional, but they do have information on whether health information is normal or not, so they can ensure that if we think you are at risk we will advise you to seek help.
- We will also keep an eye on the damp and temperature measurements in your home, and contact Leeds City Council Housing Services if these are unusual. This might result in someone from Leeds City Council getting in touch to see if they can help.

We recognise that we are asking you to do quite a lot of things in the study, and as such we would like to express our appreciation for your involvement by offering you a £20 gift voucher each time you fill in a survey, or participate in an interview.

It is quite possible that you will not gain any benefits from participating in this study, but it will help us and our colleagues at Leeds City Council to learn more about how to help people access enough heat and other energy services in the future. We hope to learn a lot more about how to improve people's lives in Leeds during the project. We also hope to use this information to create better support for people who struggle to access adequate energy in Leeds.

Ethical information about WELLBASED

The study has been approved by the University of Leeds Research Ethics Committee, which means that a committee has checked that our research plans are not going to be harmful to you. You can contact the research team if you do not understand something or if you need more information about the project or the ethical review. The names and contact details of research team members can be found under 'contacts' below.

You are invited to participate in the study because you live in a building due to be renovated by Leeds City Council. Your participation in this study is entirely voluntary and you may decide NOT to participate. This

will not affect whether the renovation project goes ahead. If you decide to participate, you can change your decision and withdraw your consent at any time. Again, this will not affect the renovation project at all. To withdraw your consent you should contact the research team (see 'contacts' below) or tell us when we get in touch with you asking for information.

What we will do with the information we collect from you

The information we collect from you will be stored by the research team in a secure location (password protected and encrypted). Some of the information will be stored in Spain, where our research partners are experts in keeping anonymous information in a safe manner. Other parts of the information will be stored in the UK. We will need to store your contact details during the project so that we can come back to you when we need to ask for further information. These contact details will be stored safely, and then deleted at the end of the project. Only the researchers on WELLBASED will have access to your contact information.

We will link each of your contributions (answer to the survey, details about temperature and damp measurements, health information etc.) using a unique code that represents you. This code replaces contact information, so that it does not include information that can directly identify you.

If you start the study, and then decide to withdraw, your consent to participate in this study, no new data will be added to the database. Once we have collected data from you, we will not be able to delete it. You can request a copy of the data whenever you want to by contacting the research team (see contacts below). You can also ask us to provide information about how we have used your data in research studies.

After the study the information will be stored in a secure location and maybe used in future research projects. Note that when the information is stored after the study, we will have deleted your contact details, and there will be no way of identifying you.

Contacts

If you would like any more information or have any questions about the research please contact us as follows:

PDRA name, University of Leeds, email, phone number.

DRAFT

Draft version of participant information leaflet and informed consent form (Leeds study site).

2. Information sheet for control group

About this information sheet

This Participant Information Sheet is intended to help you decide if you would like to participate in the WELLBASED research study. It describes the study, what you can expect to happen if you decide to take part, and contains important information to help you make your decision. Please note that:

- Participating in this study is voluntary – it is your choice;
- If you join this study, you can change your mind and withdraw at any time;
- It is important you understand why and how this study will be conducted, please read this information sheet carefully;
- The benefits and risks of your participation are also described below.

Please take time to read this information, and if you like, discuss it with friends or family to help you decide about whether you would like to participate. Once you have read this information sheet, and if you feel you have enough information and wish to participate in the project, we invite you to give your consent to being involved by signing a consent form.

About the Wellbased project in Leeds

WELLBASED is a project aimed at improving health and reducing fuel poverty in cities in Europe, including Leeds. Fuel poverty is when people find it difficult to afford their energy bills, and it can affect people's health (including mental health), for instance, when people struggle to keep their homes warm enough in winter. The project is designed to help cities like Leeds to find ways of helping people to reduce fuel poverty and reduce the effects of fuel poverty on people's health and wellbeing.

The project involves research in six European countries (Hungary, Latvia, the Netherlands, Spain, Turkey, the United Kingdom), with households that are vulnerable to fuel poverty. In each country we will run a project to improve the lives of those vulnerable to fuel poverty, and then monitor the effects of that project on people's health and wellbeing.

In Leeds we are monitoring the effects of a Leeds City Council project to renovate buildings making them more energy efficient. You do not live in one of the buildings that is currently being renovated, but your building may be renovated in the future. This is why we are inviting you to be part of our study. We want to find out more about your health and energy experiences, in order to understand what the benefits of renovation might be.

Description of the study and your involvement

In the WELLBASED study we want to understand more about your health, wellbeing and energy consumption. The key activity we would like you to be involved in is to complete a detailed questionnaire about your health, wellbeing and energy consumption on 4 separate occasions (between 01/09/2022 and 01/03/2024). We will also ask you about some key information on you and your household (e.g. age, number of occupants, income). We can help you to fill in this questionnaire if you need us to, or we can give you an internet link for you to do this yourself. This will take about 30 minutes each time and we will remind you when it needs to be done.

Risks and benefits of being in this study

Talking or answering questions about not being able to access enough energy can be upsetting, and it can also be upsetting or embarrassing to talk about your health, or your energy use with a stranger. We can make this easier for you. For instance, you can fill in the questionnaire privately online if you wish to. Information about your health and experiences will be kept private: while our research team will use the

Please note: do not make publicly available.

DRAFT

information, it will not be linked to you personally. If you are likely to be upset by thinking about and talking about these things you should probably not agree to participate in this study.

It is quite possible that you will not gain any benefits from participating in this study, but it will help us and our colleagues at Leeds City Council to learn more about how to help people access enough heat and other energy services in the future. We hope to learn a lot more about how to improve people's lives in Leeds during the project. We also hope to use this information to create better support for people who struggle to access adequate energy in Leeds.

Ethical information about WELLBASED

The study has been approved by the University of Leeds Research Ethics Committee, which means that a committee has checked that our research plans are not going to be harmful to you. You can contact the research team if you do not understand something or if you need more information about the project or the ethical review. The names and contact details of research team members can be found under 'contacts' below.

You are invited to participate in the study because you live in a building managed by Leeds City Council. Your participation in this study is entirely voluntary and you may decide NOT to participate. If you decide to participate, you can change your decision and withdraw your consent at any time. To withdraw your consent you should contact the research team (see 'contacts' below) or tell us when we get in touch with you asking for information.

What we will do with the information we collect from you

The information we collect from you will be stored by the research team in a secure location in Spain (password protected and encrypted), where our research partners are experts in keeping anonymous information in a safe manner. We will need to store your contact details during the project so that we can come back to you when we need to ask for further information. These contact details will be stored safely, and then deleted at the end of the project. Only the researchers on WELLBASED will have access to your contact information.

We will link each of your contributions using a unique code that represents you. This code replaces contact information, so that it does not include information that can directly identify you.

If you start the study, and then decide to withdraw, your consent to participate in this study, no new data will be added to the database. Once we have collected data from you, we will not be able to delete it. You can request a copy of the data whenever you want to by contacting the research team (see contacts below). You can also ask us to provide information about how we have used your data in research studies.

After the study the information will be stored in a secure location and maybe used in future research projects. Note that when the information is stored after the study, we will have deleted your contact details, and there will be no way of identifying you.

Contacts

If you would like any more information or have any questions about the research please contact us as follows:

PDRA name, University of Leeds, email, phone number.

DRAFT

Participant identifier code:

3. Consent form for intervention and control groups

If you agree with the aims of the research, consider that you have received sufficient information and wish to participate in the project, please fill in the information below and sign the document. You can withdraw your consent at any time.

I, ____ «name and surname of the participant» , declare that I have spoken to ________________________________«name and surname of the investigator» **and accordingly**: (tick as appropriate, unchecked boxes will be regarded as a refusal)

Participation in the project.

☐I express my willingness to participate in the study.

☐ I confirm that I have read and understand the information sheet dated [**insert date**] explaining the above research project and I have had the opportunity to ask questions about the project.

☐ I understand that my participation is voluntary and that I am free to withdraw at any time without giving a reason and without there being any negative consequences. In addition, should I not wish to answer any particular question or questions, I am free to decline.

Processing and storage of personal data.

☐ I authorise the research team to use my data for the purposes described in this document.

☐ I authorise the research team to store my data in an anonymised form after the completion of the project.

☐ I understand that members of the research team may have access to my anonymised responses. I understand that my name will not be linked with the research materials, and I will not be identified or identifiable in the reports that result from the research. I understand that my responses will be kept strictly confidential.

☐ I understand that the data collected from me may be stored and used in relevant future research in an anonymised form.

☐ I accept that the research team will contact me in the future if they wish to collect new data.

☐ I understand that if I withdraw from the study, the information I have already provided will not be removed from our records.

Restrictions on the use of personal information.

If you want to restrict the use of your information in any additional way, please let us know here. This may mean that we cannot include you in the research.

☐ I wish to include the following restriction on the use of my data:

Support needed to engage in the questionnaire

The survey part of the study will require you to use the internet to fill in a questionnaire at regular intervals. Please let us know our needs:

☐ I am happy to complete the questionnaires online on my own

☐ I will need help from a researcher to complete the questionnaires

☐ I do not have internet access

Accessing information about me in the study

You have the right to access information generated about you in the study. If you wish to do so you should indicate below:

Please note: do not make publicly available.

DRAFT

Draft version of participant information leaflet and informed consent form (Leeds study site).

☐ Yes, I wish to obtain the results of the research that are relevant to me.

☐I do not wish to receive information.

Name:

Date:

Signature:

(Handwritten or in legally valid electronic format)

DRAFT

Please note: do not make publicly available.

Draft version of participant information leaflet and informed consent form (Leeds study site).

4. Data Privacy Notice for intervention and control groups.

Why are we collecting your personal information?
In order to achieve the objectives of our research, we need to collect your personal information (name and contact details) for the following purposes:

- to manage the informed consent procedure for participation in the WELLBASED project.
- to allow you to participate in research activities
- for our administrative management of the project.

Who is the data controller?
They responsible institutions or organisations are:
University of Leeds, Leeds, LS29JT

How can you contact our data protection officer?
The Data Protection Officer is the person who is responsible for monitoring our compliance with data protection laws and assisting you. If you have any questions or queries about how we process your data, you should contact the lead researcher on WELLBASED:

What is the legal basis under which we process your personal information?
The data will be processed by the members of the research project under:
▪ Consent of the person concerned to participate in the research (article 6.1.a) GDPR).
▪ Legitimate interest/ task carried out in the public interest in the disclosure of press releases, news or activity reports of images or personal data when the right to information is involved (article 6.1.e) and f) GDPR).

Who are the recipients of your personal data?
Your personal data will be stored by the research team only.

How long do we keep your data?
Personal data will be kept for the life of the project (until February 2024).
Irreversibly anonymized information will be kept for research purposes for an indefinite period of time and may be shared with other entities for these purposes. You will not be identifiable in this information.

How do we protect your information?
Your information will be stored in password protected, encrypted files.

How can you exercise your rights?
In order to maintain control over your data at all times, you have the right to access your personal information, as well as to request the rectification of inaccurate data or, where appropriate, request its cancellation or deletion. Under certain circumstances, and for reasons related to your particular situation, you may object to the processing of your data. Similarly, you may exercise your right to restriction of processing of your personal information, requesting its conservation, and also the right to the portability of your data. The exercise of rights is personal and therefore we need to identify you unequivocally. You can exercise your rights in two ways:

1. By sending an email to this address: Please write to us from the email address that we have on file for you.
2. By sending a letter to us directly or by post to:

You may need to provide supporting documentation:

Please note: do not make publicly available.

DRAFT

- Proof of identity such as drivers licence or passport, including your full name;
- Details of what you want us to do with your data;
- Your address, and proof of address;

Please date and sign any correspondence you have with us.

Who can you complain to?
If you wish to complain about the processing of your personal data, you can contact the University of Leeds data protection officer: dpo@leeds.ac.uk

Additional information.
In application of the FAIR principles, data obtained in research funded by the Horizon 2020 Programme must be Findable, Accessible, Interoperable and Reusable. To meet the goal of reusability WELLBASED PROJECT will:

- Irreversibly anonymise personal data at the end of the research.
- Store anonymised data in a way that allows other researchers to access it, and use it in the future.

5. Withdrawal of consent for intervention and control groups

I revoke the consent given on the date _____________to participate in the WELLBASED project.

Full name of the participant:

Signature:

Date:

DRAFT
